# Enabling digital multifactorial risk assessment in primary care: an umbrella review and recommendations for design and implementation

**DOI:** 10.1136/bmjhci-2025-101896

**Published:** 2026-03-03

**Authors:** Lily C Taylor, Niels Peek, Ari Ercole, Georgios Lyratzopoulos, Juliet A Usher-Smith

**Affiliations:** 1 The Primary Care Unit, Department of Public Health and Primary Care, University of Cambridge, Cambridge, UK; 2 The Healthcare Improvement Studies (THIS) Institute, Department of Public Health and Primary Care, University of Cambridge, Cambridge, UK; 3 Cambridge University Hospitals NHS Foundation Trust, Cambridge, UK; 4 Epidemiology of Cancer Healthcare and Outcomes (ECHO), Department of Behavioural Science and Health, Institute of Epidemiology and Health Care (IEHC), University College London, London, UK

**Keywords:** BMJ Health Informatics, Primary Health Care

## Abstract

**Objectives:**

To develop recommendations to inform development and integration of predictive digital health and artificial intelligence tools in primary care.

**Methods:**

Recommendation development involved two stages. The initial scoping phase comprised an umbrella review to identify barriers to implementation for risk prediction tools in primary care. The consensus phase involved a stakeholder workshop with 22 stakeholders. The draft recommendations were then refined via a stakeholder survey completed by 13 participants and three online meetings attended by 14 individuals to generate the final output.

**Results:**

The umbrella review included 12 reviews and identified 15 barriers to implementation of risk prediction models, including lack of integration with electronic health records and poor interoperability across them. The final recommendations include 14 core features of risk prediction models and tools, including the need for codesign with clinicians and the public and integration with digital infrastructure and workflows.

**Discussion:**

These findings particularly emphasise the value of early engagement with key stakeholders and health record system providers, and a need for shared understanding of the needs of end-users.

**Conclusions:**

We have developed recommendations detailing 14 key characteristics for a digital risk prediction model to be successfully used in primary care settings. This profile should be used to guide development of new risk prediction tools and is also applicable more widely to other digital health innovations within primary care. Future research should work to resolve the identified system-level barriers to implementation.

**WHAT IS ALREADY KNOWN ON THIS TOPIC**

- Implementing risk prediction tools into primary care faces complex and distinct barriers with many such tools being developed and comparatively few being successfully adopted into practice.

**WHAT THIS STUDY ADDS**

- This study synthesises the existing evidence on barriers to implementation of predictive algorithms and tools in primary care and develops recommendations to summarise and guide the necessary characteristics to optimise regulatory approval and adoption.

**HOW THIS STUDY MIGHT AFFECT RESEARCH, PRACTICE OR POLICY**

- Stakeholders involved in developing and implementing predictive tools in primary care, including developers, researchers, regulators and industry representatives, should use these recommendations to produce algorithms and tools which are compatible with routine use in general practice.

## Introduction

 Development of clinical risk prediction models has rapidly increased over recent years.^1^ However, few have been successfully implemented in clinical practice, despite existing guidance for development and validation, increasing healthcare policy focus and the potential benefits of artificial intelligence (AI).^2^,^4^ The 10-Year Health Plan for England emphasises three shifts: (1) from hospital to community; (2) from analogue to digital and (3) from sickness to prevention.^5^ Understanding of barriers to implementing risk prediction models within primary care and requirements for use is urgently needed if this vision of digitally enabled health in the community is to be meaningfully realised.^5^ Therefore, we sought to develop recommendations to inform development and integration of predictive digital health and AI interventions within primary care, aimed at all stakeholders involved in developing and implementing predictive tools in UK primary care including developers, researchers, regulators and industry representatives.

## Methods

Target Product Profiles are increasingly used in healthcare to operationalise the needs of stakeholders to target and guide development.^6 7^ They summarise necessary product characteristics and have been shown to accelerate regulatory approval.^7 8^ We used guidance from the Cancer Research UK ‘Advancing the development and use of diagnostic target product profiles for cancer’ report to support development of our recommendations.^9^ Key elements include: (1) a scoping phase; (2) drafting phase and (3) consensus building.^9^ Due to the maturity of the field, we conducted an umbrella review as our scoping phase, broadly guided by the Cochrane handbook for Overviews of Reviews.^10^ We incorporated consultation with key stakeholders, including two patient and public representatives, via an online survey and three online meetings during the consensus phase.

### Scoping phase

#### Search strategy

We searched PubMed/MEDLINE electronic database using title and abstract search terms (“risk” AND “primary care”) and MeSH terms (“Medical Informatics”, “Artificial Intelligence” and “Primary Care”) up to March 2025. We restricted the search date to the last ten years (i.e. from March 2015 onwards) to capture contemporary citations considering recent advancements in risk prediction and AI.

#### Study selection

We defined multifactorial risk assessment as including two or more individual-level risk factors beyond age and sex, including phenotypic or genetic factors. Peer-reviewed English language studies fulfilling the following criteria were eligible for inclusion:

- Were systematic, scoping or meta-reviews;
- Included studies of real-time implementation of digital tools for multifactorial risk assessment, at least one of which used risk assessment to estimate the risk of undiagnosed disease in symptomatic patients or the risk of future disease in asymptomatic patients;
- Included at least one study within primary care.

Review articles without a systematic search, asynchronous multifactorial risk assessment, or risk assessment exclusively outside of primary care, and studies focused on development, validation or impact evaluation of risk prediction models were excluded.

Supported by an information specialist, one reviewer (LCT) completed database searching, de-duplication and title and abstract review. A second reviewer (JAU-S) independently screened 10% of citations, resolving discrepancies together. A single reviewer (LCT) completed full text review for all citations, and a second reviewer (JAU-S) screened 20%. The reference lists of all eligible citations were reviewed to identify additional papers.

#### Data extraction and synthesis

Two reviewers (LCT, JAU-S, AE, NP or GL) conducted data extraction using a standardised form. We analysed the findings through codebook thematic analysis using the four domains of the Engineering Better Care systems-based approach as our initial codebook: (1) People; (2) Systems; (3) Design and (4) Risk.^11 12^ This framework has previously been used to explore stakeholder views, map healthcare pathways and identify systems-level solutions and was chosen to help identify where the barriers to implementation lie.^13 14^ We reviewed the identified barriers to implementation and generated key themes and associated descriptors. This was an iterative process led by the first author (LCT) and supported by a second researcher (JAU-S).

#### Quality assessment

A single reviewer (LCT) conducted quality assessment for all citations using the Risk Of Bias In Systematic reviews (ROBIS) tool, specifically designed for critical appraisal of systematic reviews.^15^ A second reviewer (JAU-S) completed quality assessment for 25% of citations.

### Consensus phase

#### Stakeholder workshop

Review findings were presented at a 1-day workshop in June 2025 with 22 stakeholders from primary care, medical informatics, data innovation and commercialisation, integrated care system IT procurement, and the Medicines and Healthcare products Regulatory Agency recruited through the authors’ personal networks via email. This was followed by a group discussion which was narratively summarised and used to develop core features required for successful implementation of risk prediction tools in primary care. These core features were mapped onto the Target Product Profile categories.^9^

#### Stakeholder consensus building

The final stage was establishing consensus with stakeholders. Workshop attendees were invited to complete an anonymous online survey on the initial profile draft (online supplemental table 1). They were asked to what extent they agreed with the core features (‘strongly disagree-strongly agree’) with 80% agreement considered a consensus, and whether there was anything essential missing. Survey results and the final recommendations were refined at two 1-hour online meetings in August 2025. The results were also shared with two patient and public involvement (PPI) members in an online meeting to gain their feedback. Three researchers (NP, JAU-S and LCT) with relevant topical and methodological expertise facilitated the consensus elements and abstained from taking part.

#### Refining the recommendations

In phase 1, we converted each barrier into a need, merged those that were comparable or highly related (eg, concerns relating to analytic performance, clinical validity and utility were inter-related) to generate a draft (online supplemental table 2a). In phase 2, we incorporated additional factors identified in the workshop discussion (online supplemental table 2b). Finally, we used a survey and online consensus discussions to refine the recommendations.

## Results

### Study selection

**Figure 1:**
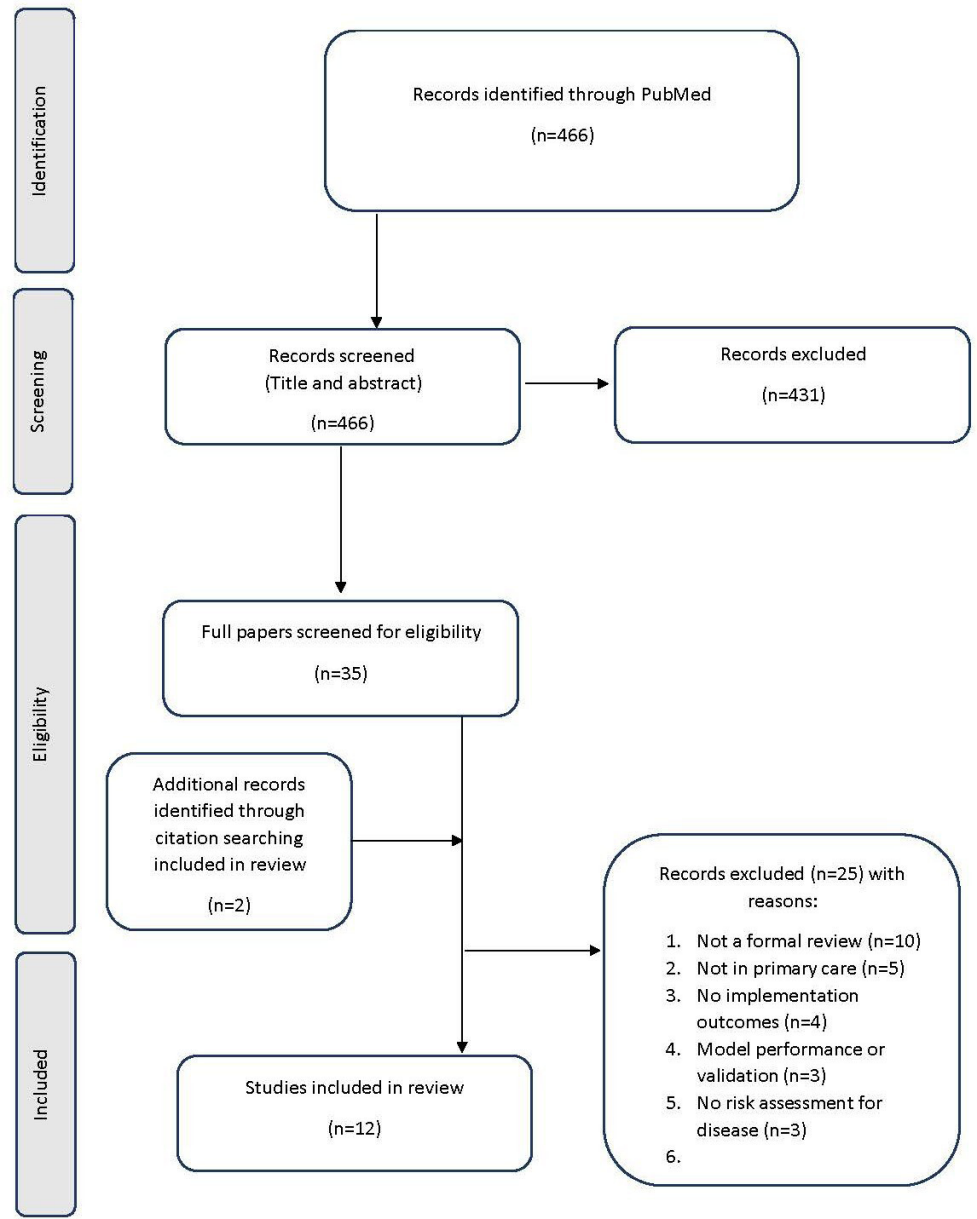
PRISMA flow diagram. PRISMA, Preferred Reporting Items for Systematic Reviews and Meta-Analyses.

The search generated 466 citations. 431 citations were excluded following title and abstract review with 97.9% agreement. An additional 25 papers were excluded following full text review (figure 1). Two further citations were identified via reference list searching.

### Review characteristics

Review characteristics are summarised in online supplemental table 3. Just over half pertained specifically to primary care (n=7/12, 70%).^16^,^22^ All reviews focused primarily on perspectives of healthcare professionals (HCPs) as the end user, except for one, He *et al*, which focused on patients.^19^

### Quality assessment

Most reviews were of high quality (9/12, 75%),^17^,^2023^ two reviews were of uncertain quality (2/12, 17%)^21 27^ and a single review was at high risk of bias (1/12, 8%)^22^ (online supplemental table 3).

### Barriers identified

**Figure 2:**
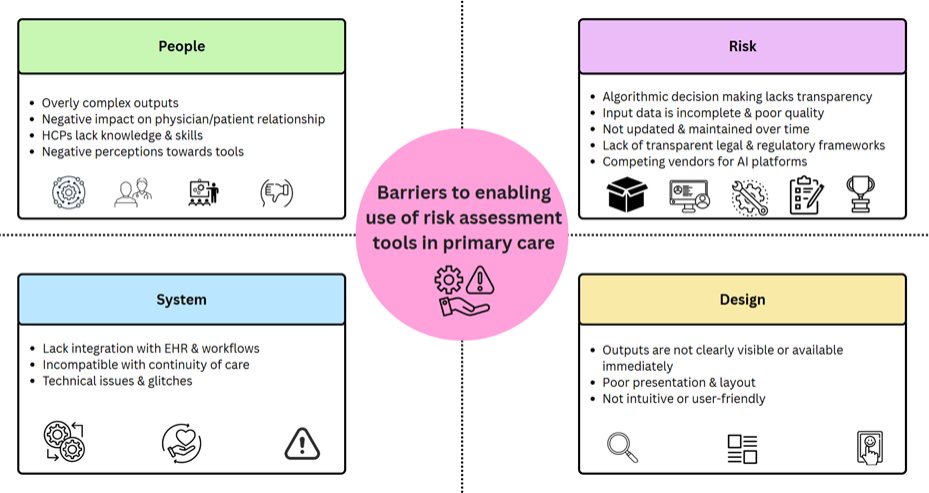
Summary of the barriers to enabling the use of risk assessment tools in primary care categorised according to the systems-based approach. AI, artificial intelligence; EHR, electronic health records; HCP, healthcare professional.

15 barriers were identified and all were included by at least two reviews (online supplemental table 4, figure 2). No reviews included all 15 barriers.^16^,^1820^

### Thematic analysis

The barriers are summarised according to the domains of the systems approach (figure 2).

#### The people domain

This domain covers the perspectives of people using and working within the system.

##### Tools have overly complex outputs

This barrier was reported by two reviews (2/12, 17%).^19 24^ Risk prediction tools with overly complex outputs were considered unsuitable for patients with low literacy or language skills,^19 24^ inhibiting their use in shared decision-making.^24^

##### Negative impact on the patient/physician relationship

This barrier was reported by six reviews (6/12, 50%). Use of tools within consultations was considered intrusive and disruptive, hindering physician/patient interactions.^17^,^2023 25^

##### HCPs lack knowledge and skills

HCPs’ lack of knowledge and skills was reported by seven reviews (7/12, 58%).^18 20 21 23 24 26 27^ Clinicians reported poor understanding of AI and medical informatics and lacked relevant experience and skills.^21 26^ It was acknowledged that HCPs required greater training to implement tools safely and effectively.^18 20 21 23 24 27^ Two reviews reported that HCPs were unaware of or did not understand the purpose of predictive tools, representing a barrier to engagement.^20 26^

##### Negative perceptions towards risk prediction tools

Most citations reported negative perceptions towards risk prediction tools (9/12, 75%), including HCP^16^,^1820 23 25^ and patient opinions.^24^ HCPs perceived tools as time-consuming, burdensome and unnecessary, stating poor trust in their outputs.^16^,^1820 23 24^ Specifically, HCPs were sceptical about accounting for multimorbidity and felt that the recommendations lacked a holistic perspective.^23 25 27^ For GPs, use of predictive tools negatively impacted their role as gatekeepers, and they had concerns about potential over-referrals.^16^ Some HCPs felt ‘marked down’ by an external authority infringing on their sense of autonomy when the tool’s recommendations conflicted with their own.^16 18 20 25 26^

### Systems domain

This domain covers interconnected sociotechnical elements of the system.

#### Lack of integration with electronic health records and workflows

The most commonly reported barrier was predictive tools lacking integration with electronic health records (EHRs) and clinical workflows (11/12, 92%).^16^,^1820^ Poor integration and insufficient automation caused HCPs to rely on manual data entry which was viewed as error-prone and time-consuming.^1820 22 25^,^27^ Poor interoperability contributed to conflicts with technical and clinical workflows.^27^ A further major barrier was alert fatigue.^16^,^1820 21 25 27^ Excessive pop-ups generated additional tasks in time-constrained settings and appeared at cognitively inopportune times in clinical workflows.^16^,^1820^

#### Incompatible with continuity of care

A further barrier was incompatibility with continuity of care, cited by two reviews (2/12, 17%).^18 19^ Tools not well-designed or integrated to enable multidisciplinary care and those that do not facilitate data sharing between primary care providers or with secondary care services hindered use.^18 19^

#### Technical issues and glitches

Technical issues and glitches were reported in four reviews (4/12, 33%).^18 20 25 27^ Tools were perceived as slow or glitchy and IT hardware was often lacking.^18 20 25^ Similarly, digital infrastructure was inconsistent across healthcare settings, limiting usage.^20 27^

### Design domain

This domain identifies design challenges that need to be addressed to deliver appropriate outcomes.

#### Outputs are not clearly visible or available immediately.

Participants in two studies (2/12, 17%) reported that recommendations from clinical tools are not always clearly visible or immediately available, preventing actionability within a consultation, thereby reducing overall utility.^23 25^

#### Poor presentation and layout

Poor presentation and layout of outputs was reported by six reviews (6/12, 50%).^18^,^2023^ Outputs were considered visually unappealing, lacking important information and badly formatted, discouraging adoption.^18^,^2023^ Numerical data was poorly presented and often required graphical representations or visual aids that were not available.^19 20 24^

#### Not intuitive or user-friendly

Four reviews reported that tools are not user-friendly or intuitive, which disincentivised their use (4/12, 33%).^18 22 23 25^

### Risk domain

This domain involves identification of risks or threats within the system and highlights areas of uncertainty or unintended consequences.

#### Algorithmic decision-making lacks transparency.

Four reviews reported concerns over validity, reliability, accuracy and security of algorithms and AI (4/12, 33%).^16 20 21 27^ Lack of clarity around algorithmic decision-making processes contributed to these concerns, with participants conceptualising the algorithm as a ‘black box’ with potential for algorithmic bias.^16 20 27^

#### Input data are incomplete and poor quality

Concerns over poor quality and low quantity of input data were reported across three reviews (3/12, 25%).^18 25 27^ Data in the EHR were described as being of low quality and accuracy, and algorithms trained on datasets of limited size.^18 27^ This contributed to fears that they have low validity across populations and are subject to distributional shift and automation bias, exacerbating demographic inequalities.^27^

#### Not updated and maintained over time

Participants across three reviews reported concerns that tools are not regularly maintained or updated (3/12, 25%).^20 22 26^ Participants felt that the implementation of tools should be iterative and improvements made in consultation with HCPs and patients as end-users.^20 26^

#### Lack of transparent legal and regulatory frameworks

Three reviews mentioned a lack of transparent regulation and ambiguous legal implications (3/12, 25%).^19 26 27^ Specifically, participants reported unclear liability for legal risks, poorly defined ownership and governance of data and concerns over privacy, security and consent.^19 26 27^

#### Competing vendors for AI platforms

A single review reported competition between vendors for AI platforms (1/12, 8%).^27^ This included concerns that AI vendors are motivated by profit rather than patient outcomes, potentially lowering patient safety and reducing acceptability.^27^

### Recommendations for design and implementation of digital tools for multifactorial risk assessment

We used the review findings in combination with stakeholder consensus meetings attended by 14 participants and an online survey completed by 13 individuals to develop a set of recommendations of design and implementation (table 1). The initial list was amended according to stakeholder feedback to generate the final 14 key characteristics covering 8 prespecified domains. Cost and economic considerations did not feature in the recommendations due to the lack of a specific use case.

**Table 1:** Final recommendations for design and implementation of multifactorial risk prediction tools in primary care

Target product profile category	Core feature for a risk prediction tool
Unmet need	Tools should be codesigned alongside a range of relevant clinicians and members of the public to ensure they address a strategic clinical need and align with policy priorities.
Analytical performance	Algorithms should demonstrate sufficient predictive performance and undergo validity testing ahead of implementation. Developers should be transparent about the impact of missing or poor-quality input data on the uncertainty and accuracy of the tool’s outputs. The development and validation of risk prediction models should be reported transparently, in line with established guidelines, e.g. TRIPOD+AI.^28^ Tools must be able to demonstrate clinical effectiveness in real-world contexts and document workflow impact and unintended consequences. Tools must be monitored, maintained and updated over time to track automation bias, mitigate distributional shift and ensure ongoing clinical effectiveness.
Clinical validity	
Clinical utility	
Human factors	Tools should be designed for intuitive integration with clinical workflows to avoid disrupting the physician/patient relationship. HCPs should receive training and education before implementation to facilitate routine use and alleviate negative perceptions. If patient-facing, recommendations/outputs should be co-designed with the public and clinicians to ensure they are patient-oriented and accessible, in line with established guidelines, for example, Double Diamond^29^ and Web Content Accessibility Guidelines.^32^
Infrastructure	Tools should strive to be integrated with EHR and incumbent technology. Tools should strive to be interoperable using standardised taxonomy. There should be ongoing access to technical support for users and opportunity for users to provide feedback.
Cost and economic considerations	
Regulation	Tools must demonstrate compliance with legal, regulatory and data protection frameworks to facilitate trust and confidence, for example, MHRA guidance on regulating medical devices.^33^
Environmental impact	Tools should incorporate efficient algorithms and make use of shared compute resources to minimise environmental burden

Black text—derived from the umbrella review findings; blue text—derived from the stakeholder workshop; green text—derived from the stakeholder survey and online meetings.

AI, artificial intelligence; EHR, electronic medical record; HCPs, healthcare professionals; MHRA, Medicines and Healthcare product Regulatory Agency; TRIPOD, Transparent Reporting of a multivariable prediction model for Individual Prognosis or Diagnosis.

## Discussion

To our knowledge, this is the first umbrella review of barriers to implementation of real-time multifactorial risk assessment tools in primary care and the first set of recommendations for such tools. Twelve reviews were included, enabling us to identify 15 barriers to implementation. Common barriers included a lack of integration and interoperability, and negative perceptions from HCPs. We identified 14 desired features of digital risk prediction models to enable successful integration. For several features, we signpost existing guidelines that may be used to implement our recommendations, for example, Transparent Reporting of a multivariable prediction model for Individual Prognosis or Diagnosis (TRIPOD)+AI and Double Diamond.^28 29^

The first feature is that tools address an unmet need, highlighting the importance of codesign with clinicians and the public to ensure fulfilment of unmet needs that have clinical relevance aligned with policy priorities, and that the outputs and integration of such tools are tailored to end-users. Predictive tools should enable integration with EHRs and clinical systems by adopting standards for terminology and health information exchange. Risk assessment tools should also seek to minimise environmental burden wherever possible, for example, by striving for algorithmic efficiency. Additionally, tools should be able to demonstrate clinical effectiveness, and predictive performance in testing and real-world contexts and ensure ongoing monitoring and maintenance. Together, these features span the key stages of the AI model lifecycle (requirements, design, development, validation, real-world deployment and ongoing monitoring), ensuring sustained clinical utility as populations and healthcare contexts change over time. In practice, there is a likelihood that these objectives may be in competition, necessitating trade-offs in line with specific use cases. Similarly, auditing procedures and whether minimum or optimum thresholds are most appropriate should be guided by the intervention in question.

### Comparison with existing literature

The scoping phase ensured that the features described in our final recommendations are consistent with previously published literature. Several of the reviews in this synthesis include recommendations for development and implementation of clinical support tools.^20 25^ However, none of the included reviews comprised all the identified barriers to implementation; therefore, a strength of this work lies in the synthesis of previous findings and being guided by the Target Product Profile framework. We also used stakeholder feedback to further refine our findings. An additional review by Derksen *et al*, has been published outside of the literature search dates.^30^ Our findings remain consistent with this publication which reflects the complex and intersectional barriers to implementation. Similarly, Derksen *et al*’s findings are congruent with our recommendations, including integration with digital technologies and existing workflows. However, core factors reported here are absent from their findings, including codesign with clinicians and the public, and environmental impacts, which is particularly pertinent given the rise of AI.^30^ We also highlight the importance of robust development and validation and acknowledge the existence of established guidelines to achieve this. For example, the TRIPOD+AI statement, a reporting standard for developing or validating diagnostic or prognostic prediction models, promoting transparency around the performance and validation of predictive algorithms and tools.^28^ While TRIPOD+AI seeks to ensure methodological rigour during development and validation of risk algorithms, these recommendations seek to provide core features to strive towards during development, implementation, and sustainability.

### Strengths and limitations

A key strength is use of robust systematic review methods in the scoping phase. We used two pre-established frameworks to systematise our findings in a transparent manner and were guided by Target Product Profiles and the Cochrane handbook. Not limiting to a single disease area or risk prediction algorithm enabled us to develop recommendations with a broad use case. Similarly, the breadth of studies included in the review means that the identified barriers likely apply to other digital tools used in primary care (like Clinical Decision Support Systems (CDSS) and decision support tools). Therefore, a strength is that these recommendations can be used more widely and easily adapted to related use cases. Furthermore, we included a wide range of stakeholders in the consensus phase.

This work also has several limitations. First, some characteristics cannot be defined in greater detail until a specific product is identified. Similarly, some recommendations cannot be fulfilled immediately in the development process. To acknowledge this, we have incorporated statements that the tool ‘should strive to’ fulfil. Additionally, the umbrella review search strategy was based only on key words and may have missed some articles. We also identified several reviews that were not exclusive to digital risk assessment tools, with many including CDSS not based on risk assessment. It was not possible to separate the findings relating to non-digital tools or CDSS without risk prediction. However, due to the refinement of our results via the workshop and consensus meetings which focused on digital risk assessment tools, we feel this is unlikely to have had a negative impact on the applicability of our recommendations to these tools. Finally, the subjectivity of input sources may bias the results towards certain stakeholders, as has previously been reported.^31^ However, we included stakeholders from across many other areas in the workshop, survey, and online meetings and presented the recommendations to two PPI members to gain their perspectives.

### Implications

These findings provide important insights into the requirements for successful development and implementation of digital risk prediction tools in primary care. Although primarily developed for risk prediction tools, the recommendations have wider applicability and implications for similar tools like CDSS or decision aids. We identified multiple systems-level barriers to implementation. In future, we encourage greater collaboration between policymakers, technology developers, researchers and regulators to resolve these issues and facilitate wider adoption and sustained usage. The use of the Target Product Profile categories means that this list could provide a useful foundation for generating a profile once a specific use case is identified.

## Conclusions

We have identified 14 core features of a successful digital risk prediction tool for use in primary care. These include codesign with clinicians and the public, greater integration with EHRs and interoperability, and improved transparency of underlying algorithms.

## Supplementary material

10.1136/bmjhci-2025-101896 online supplemental file 1

## Data Availability

All data relevant to the study are included in the article or uploaded as supplementary information.
