# Supplementary material for: Enabling digital multifactorial risk assessment in primary care: an umbrella review and recommendations for design and implementation
Source: BMJ Health Care Inform. 2026 Mar 3;33(1):e101896. doi: 10.1136/bmjhci-2025-101896 (PMC12958886; doi:10.1136/bmjhci-2025-101896)
Supplement: online supplemental file 1 [file bmjhci-33-1-s001.pdf]

Enabling digital multifactorial risk assessment in primary care: an umbrella review and recommendations for design and implementation: Supplementary materials

Supplementary Table 1

Anonymous consensus survey outline

Real time multifactorial risk assessment tools in primary care		
**Unmet need: The unmet need a tool should respond to and its application scope**	Tools should be co-designed alongside clinicians and patient to ensure they address a relevant clinical need and align with policy priorities	• 5-point Likert scale “Strongly disagree – Strongly agree”
	Is there anything essential missing from the unmet need section?	• Free text
**Analytical performance, clinical validity and utility: Requirements related to accuracy, validity and utility**	Algorithms should demonstrate sufficient predictive performance and undergo validity testing ahead of implementation	• 5-point Likert scale “Strongly disagree – Strongly agree”
	Developers should be transparent about the impact of missing or poor-quality data on the uncertainty or accuracy of recommendations	• 5-point Likert scale “Strongly disagree – Strongly agree”
	Algorithmic functions should be transparent to address concerns about validity, reliability, accuracy and security	• 5-point Likert scale “Strongly disagree – Strongly agree”
	Tools must demonstrate clinical effectiveness in real-world contexts and address workflow impact and unintended consequences	• 5-point Likert scale “Strongly disagree – Strongly agree”
	Tools must be monitored, maintained, and updated over time to mitigate distributional shift and automation bias and ensure ongoing clinical effectiveness	• 5-point Likert scale “Strongly disagree – Strongly agree”

	Is there anything essential missing from the analytical performance, clinical validity and utility section?	• Free text
**Human factors: Requirements relating to individuals' interaction with the tool**	Tools must be intuitively integrated with clinical workflows, with minimal user input to avoid disrupting the physician/patient relationship	• 5-point Likert scale "Strongly disagree – Strongly agree"
	HCPs should receive training and education before implementation to facilitate routine use & alleviate negative perceptions	• 5-point Likert scale "Strongly disagree – Strongly agree"
	Recommendations/outputs should be co-designed with patients and clinicians to ensure they are patient-oriented, accessible for patients with low literacy or English-language skills & are not unnecessarily complex or burdensome	• 5-point Likert scale "Strongly disagree – Strongly agree"
	Is there anything essential missing from the human factors section?	• Free text
**Infrastructure: Requirements related to facilities, equipment, supplies, IT systems or other operating conditions that need to be established and maintained**	Tools must be integrated with EMR & interoperable using standardised data elements for multi-disciplinary workflows	• 5-point Likert scale "Strongly disagree – Strongly agree"
	There should be ongoing access to technical support for users	• 5-point Likert scale "Strongly disagree – Strongly agree"
	Is there anything essential missing from the infrastructure section?	• Free text
**Cost and economic considerations: Requirements related to economic and commercial matters**	Vendors should have to adhere to a minimum set of legal and regulatory requirements to ensure patient safety is maintained despite financial motivations	• 5-point Likert scale "Strongly disagree – Strongly agree"
	Remuneration at the practice level should be considered to facilitate adoption	• 5-point Likert scale "Strongly disagree – Strongly agree"
	There should be a business case for scaling and sustainability of tools	• 5-point Likert scale "Strongly disagree – Strongly agree"

	Is there anything essential missing from the cost and economic considerations section?	• Free text
**Regulation: Features related to regulatory requirements**	Tools must adhere to legal and regulatory frameworks and data protection	• 5-point Likert scale “Strongly disagree – Strongly agree”
	Is there anything essential missing from the regulation section?	• Free text
**Environmental impact: Requirements about the tool’s environmental impact**	Tools need to incorporate efficient algorithms, be task specific and make use of shared compute resources to minimise environmental burden	• 5-point Likert scale “Strongly disagree – Strongly agree”
	Is there anything essential missing from the environmental impact section?	• Free text
End of survey		

Supplementary Table 2a

Phase 1: Review findings

Target Product Profile category	Core feature for a risk prediction tool
Unmet need	• Tools should be co-designed alongside clinicians to ensure they address a relevant clinical need
Analytical performance	• Algorithms should demonstrate sufficient predictive performance and undergo validity testing ahead of implementation<br>• Developers should be transparent about the impact of missing or poor-quality data on the accuracy of recommendations<br>• Algorithmic functions should be transparent to address concerns about validity, reliability, accuracy and security<br>• Tools must be monitored, maintained, and updated over time to mitigate distributional shift and automation bias
Clinical validity	
Clinical utility	
Human factors	• Tools must be intuitively integrated with clinical workflows, with minimal user input to avoid disrupting the physician/patient relationship<br>• HCPs should receive training and education before implementation to facilitate routine use & alleviate negative perceptions<br>• Recommendations/outputs should be co-designed with patients to ensure they are patient-oriented, accessible for patients with low literacy or English-language skills & are not unnecessarily complex or burdensome
Infrastructure	• Tools must be integrated with EMR & interoperable for multi-disciplinary workflows<br>• There should be ongoing access to technical support for users
Cost and economic considerations	• Vendors should have to adhere to a minimum set of legal and regulatory requirements to ensure patient safety is maintained despite financial motivations<br>• Remuneration at the practice level should be considered to facilitate adoption
Regulation	• Transparent legal and regulatory frameworks must be developed at a national level with clear liability for error
Environmental impact	

Supplementary Table 2b

Phase 2: Review findings + workshop findings

Target Product Profile category	Core feature for a risk prediction tool
Unmet need	• Tools should be co-designed alongside clinicians and patients to ensure they address a relevant clinical need and align with policy priorities
Analytical performance<br>Clinical validity<br>Clinical utility	• Algorithms should demonstrate sufficient predictive performance and undergo validity testing ahead of implementation<br>• Developers should be transparent about the impact of missing or poor-quality data on the uncertainty and accuracy of recommendations<br>• Algorithmic functions should be transparent to address concerns about validity, reliability, accuracy and security<br>• Tools must demonstrate clinical effectiveness in real-world contexts and address workflow impact and unintended consequences<br>• Tools must be monitored, maintained, and updated over time to mitigate distributional shift, automation bias and ensure ongoing clinical effectiveness
Human factors	• Tools must be intuitively integrated with clinical workflows, with minimal user input to avoid disrupting the physician/patient relationship<br>• HCPs should receive training and education before implementation to facilitate routine use & alleviate negative perceptions<br>• Recommendations/outputs should be co-designed with patients and clinicians to ensure they are patient-oriented, accessible for patients with low literacy or English-language skills & are not unnecessarily complex or burdensome
Infrastructure	• Tools must be integrated with EMR & interoperable using standardised data elements for multi-disciplinary workflows<br>• There should be ongoing access to technical support for users
Cost and economic considerations	• Vendors should have to adhere to a minimum set of legal and regulatory requirements to ensure patient safety is maintained despite financial motivations<br>• Remuneration at the practice level should be considered to facilitate adoption<br>• There should be a business case for scaling and sustainability of tools
Regulation	• Tools must adhere to legal and regulatory frameworks and data protection
Environmental impact	• Tools need to incorporate efficient algorithms, be task specific and make use of shared compute resources to minimise environmental burden

Supplementary Table 3

ACCORD (Accurate Consensus Reporting Document) checklist:

Item number	Manuscript section	Item wording	Page
T1	Title	Identify the article as reporting a consensus exercise and state the consensus methods used in the title.	N/A
I1	Introduction	Explain why a consensus exercise was chosen over other approaches.	Pg 5
I2	Introduction	State the aim of the consensus exercise, including its intended audience and geographical scope (national, regional, global).	Pg 4
I3	Introduction	If the consensus exercise is an update of an existing document, state why an update is needed, and provide the citation for the original document.	N/A
M1	Methods > Registration	If the study or protocol was prospectively registered, state the registration platform and provide a link. If the exercise was not registered, this should be stated.	Not registered
M2	Methods > Selection of SC and/or panelists	Describe the role(s) and areas of expertise or experience of those directing the consensus exercise.	Pg 7
M3	Methods > Selection of SC and/or panelists	Explain the criteria for panelist inclusion and the rationale for panelist numbers. State who was responsible for panelist selection.	Pg 7
M4	Methods > Selection of SC and/or panelists	Describe the recruitment process (how panelists were invited to participate)	Pg 7
M5	Methods > Selection of SC and/or panelists	Describe the role of any members of the public, patients, or carers in the different steps of the study.	Pg 7
M6	Methods > Preparatory research	Describe how information was obtained prior to generating items or other materials used during the consensus exercise.	Pg 5/6

M7	Methods > Preparatory research	Describe any systematic literature search in detail, including the search strategy and dates of search or the citation if published already.	Pg 5/6
M8	Methods > Preparatory Research	Describe how any existing scientific evidence was summarized and if this evidence was provided to the panelists.	Pg 6/7
M9	Methods > Assessing consensus	Describe the methods used and steps taken to gather panelists input and reach consensus (for example, Delphi, RAND/UCLA, nominal group technique).	Pg 7
M10	Methods > Assessing consensus	Describe how each question or statement was presented and the response options. State whether panelists were able to or required to explain their responses, and whether they could propose new items.	Supplement
M11	Methods > Assessing consensus	State the objective of each consensus step.	Pg 8
M12	Methods > Assessing consensus	State the definition of consensus (for example, number, percentage, or categorical rating, such as “agree” or “strongly agree” and explain the rationale for that decision.	Pg 7
M13	Methods > Assessing consensus	State whether items that met the prespecified definition of consensus were included in any subsequent voting rounds.	Pg 7
M14	Methods > Assessing consensus	For each step, describe responses were collected, and whether responses were collected in a group setting or individually.	Pg 7
M15	Methods > Assessing consensus	Describe how responses were processed or synthesised.	Pg 8
M16	Methods > Assessing consensus	Describe any piloting of the study materials and/or survey instruments.	N/A

M17	Methods > Assessing consensus	If applicable, describe how feedback was provided to panelists at the end of each consensus step or meeting.	Pg 8
M18	Methods > Assessing consensus	State whether anonymity was planned in the study design. Explain where and to whom it was applied and what methods were used to guarantee anonymity.	Pg 7
M19	Methods > Assessing consensus	State if the steering committee was involved in the decision made by the consensus panel.	Pg 8
M20	Methods > Participation	Describe any incentives used to encourage responses or participation in the consensus process.	N/A
M21	Methods > Participation	Describe any adaptations to make the surveys/meetings more accessible.	N/A
R1	Results	State when the consensus exercise was conducted. List the date of initiation and the time taken to complete each consensus step, analysis, and any extensions or delays in the analysis.	Pg 7
R2	Results	Explain any deviations from the study protocol, and why these were necessary.	N/A
R3	Results	For each step, report quantitative (number of panelists, response rate) and qualitative (relevant sociodemographics) data to describe the participating panelists.	Pg 23
R4	Results	Report the final outcome of the consensus process as qualitative (for example, aggregated themes from comments) and/or quantitative (for example, summary statistics, score means, medians, and/or ranges) data.	Table 3
R5	Results	List any items or topics that were modified or removed during the consensus process. Include why and when in the process they were modified or removed.	Supplement & Table 3

D1	Discussion	Discuss the methodological strengths and limitations of the consensus exercise.	Pg 27/28
D2	Discussion	Discuss whether the recommendations are consistent with any preexisting literature and, if not, propose reasons why this process may have arrived at alternative conclusions.	Pg 27
O1	Other information	List any endorsing organisations and their role.	Pg 29
O2	Other information	State any potential conflicts of interests, including among those directing the consensus study and panelists. Describe how conflicts of interest were managed.	Pg 29
O3	Other information	State any funding received and the role of the funder.	Pg 30

Supplementary Table 4

Summary of included study characteristics

Author and year:	Title:	Perspective & setting:	Primary aim:	Study design:	Number of included citations:	ROBIS result:	Author's primary conclusions:
Nurek et al., 2015[27]	Reducing diagnostic errors in primary care. A systematic meta-review of computerized diagnostic decision support systems by the LINNEAUS collaboration on patient safety in primary care	HCPs in primary care	To conduct a review of existing systematic reviews to assess the current consensus on how CDSS can meet the requirements of supporting the cognitive task of diagnosis, and the currently perceived barriers that prevent the integration of CDSS with EHR systems.	Meta-review	12	High risk of bias	IT advances may allow for the development of effective CDSS, including development of dynamic semantic tools and standardisation of knowledge representation. EHR should be considered as a knowledge-manager rather than a static system.

Kilsdonk et al., 2017[16]	Factors influencing implementation success of guideline-based clinical decision support systems: A systematic review and gaps analysis	HCPs in all healthcare settings	To support software teams in development and implementation of guideline-based CDSS.	Systematic review	35	Low risk of bias	A range of factors are associated with HCP usage of guideline-based CDSS with research gaps around organisational factors that facilitate successful implementation. Future research should evaluate implementation of CDSS using a multidimensional approach & investigate whether model usage in the planning phase aids in prevention of implementation barriers.
Chima et al., 2019[17]	Decision support tools to improve cancer diagnostic decision making in primary care: A systematic review	HCPs in primary care	To summarise existing evidence on the effects of eCDSTs on decision making for cancer diagnosis in primary care & determine factors that influence their successful implementation.	Systematic review	12	Low risk of bias	eCDSTs have the potential to improve decision making for cancer diagnosis in primary care. The optimal mode of delivery remains unclear. Advancement and sustainability relies on continuous feedback from HCPs and tool development. Improvements to implementation & integration with GP workflows are needed.
Scalia et al., 2019[23]	The impact and utility of encounter patient decision aids: Systematic review, meta-analysis and narrative synthesis	Stakeholders in all healthcare settings	1) To determine the effect associated with the use of encounter PDAs by conducting a meta-analysis of RCTs; 2) to conduct a narrative	Systematic review & meta-analysis	53	Low risk of bias	ePDAs help HCPs practice shared decision making. aPDAs have high utility across all HCPs in the clinical setting but there are concerns around tool integration. HCP input & education on using tools may improve feasibility.

			synthesis of non-randomised studies to determine the feasibility, utility & integration into clinical workflow from different stakeholder perspectives.				
Harada et al., 2021[25]	Clinical decision support systems for diagnosis in primary care: A scoping review	HCPs in primary care	To verify the usefulness of CDSS in the diagnostic domain in primary care & to identify research gaps & areas of uncertainty that require further exploration.	Scoping review	26	Unclear risk of bias	CDSS have significant results in screening for chronic diseases. Future research should focus on the utility of CDSS in other disease and clinical contexts. The combination of CDSS & AI represents a potential opportunity to improve healthcare.
Chen et al., 2022[24]	Barriers and enablers to implementing and using clinical decision support systems for chronic diseases: A qualitative systematic review and meta-aggregation	HCPs in primary care, specialist outpatient services & community care	To describe HCP experiences of implementing, using, evaluating, & sustaining EHR-based CDSS interventions for chronic disease care.	Systematic review & meta-aggregation	33	Low risk of bias	Factors affecting CDSS uptake include clinical context, user, external context, & technological factors. Recommendations for research include incorporating multi-morbidity, user involvement, & efforts to sustain CDSS uptake.

Ahmed et al., 2023[22]	A systematic review of the barriers to the implementation of artificial intelligence in healthcare	HCPs in all healthcare settings	To identify & examine barriers to implementation of AI in healthcare to produce a framework for health systems to implement AII.	Systematic review	59	Unclear risk of bias	Barriers to AI in healthcare span six areas: ethical, technological, liability & regulatory, workforce, social & patient safety barriers. Such barriers must be address to realise the potential of AI in improving population health & health systems.
Alasiri et al., 2023[18]	The role of clinical decision support systems in preventing stroke in primary care: A systematic review	HCPs in primary care	1) To identify the available decision support systems or tools that have been developed & tested to support the decision making process in primary healthcare to prevent stroke; 2) to identify any barriers or facilitators encountered during design, implementation or using CDSS; 3) to summarise the core aspects of the decision aids.	Systematic review	5	Low risk of bias	CDSS can improve primary care outcomes & prevent disease. Potential benefits range from small to moderate but designing reliable CDSS is challenging. Future research should focus on novel designs that ensure reliability.

Meunier et al., 2023[19]	Barriers and facilitators to the use of clinical decision support systems in primary care: A mixed methods systematic review	HCPs in primary care	To identify & quantify the barriers & facilitators to the use of CDSSs by PCPs.	Mixed methods systematic review	48	Low risk of bias	CDSS have potential benefits to improving safety & quality of care but lack efficiency due to increased workload. Human, technological, & organisational factors must be addressed by CDSS developers to improve usability in primary care.
He et al., 2024[20]	Perceptions of primary care patients on the use of electronic clinical decision support tools to facilitate health care: A systematic review	Patients in primary care	To summarise current evidence relating to primary care patients' perceptions & experiences on the use of eCDSTs by their clinician to provide care.	Systematic review	20	Low risk of bias	Several factors impact eCDST adoption & acceptability from the patient perspective, including requiring a holistic perspective & data privacy concerns. Future research should aim to better align eCDST delivery with patient care.
Hill et al., 2024[26]	What characteristics of clinical support system implementations lead to adoption for regular use? A scoping review	HCPs in all healthcare settings	To identify CDSS features associated with adoption into routine use & to summarise common strategies leading to successful implementation & normalised use to identify	Scoping review	53	Low risk of bias	Few examples of CDSS were available for analysis, illustrating the implementation gap. There is a need for organisational support, mixed methods implementation strategies & an iterative approach to address HCP feedback.

			strategies to inform future implementations.				
Gani et al., 2025[21]	Understanding "alert fatigue" in primary care: Qualitative systematic review of general practitioners attitudes and experiences of clinical alerts, prompts, and reminders	HCPs in primary care	To synthesise current qualitative research of GPs' attitudes towards CRs, enabling an exploration of the interacting influences on the occurrence of alert fatigue in GPs, including the deployment, design, & perceived efficacy of CRs.	Systematic review	9	Low risk of bias	CRs can enhance safety, workflow management, preventative care, & reduce cognitive overload when used correctly. Recommendations for future CRs include improved graphical design, nuanced/patient-specific content, greater HCP autonomy in if/when to use CRs, & earlier, more consistent engagement with HCPs in design & development.

CR – clinical reminder; CDSS – clinical decision support system; eCDST – electronic clinical decision support tool; ePDA – electronic patient decision aid; EHR – electronic health records; GP – general practice/practitioner; HCP – healthcare professional; IT – information technology; PCP – primary care professional; UK – United Kingdom; USA – United States of America.

Supplementary Table 5

Summary of the implementation barriers identified across the included studies.

**Barrier to implementation**	**Description**	Nurek et al., 2015	Kilsdonk et al., 2017	Chima et al., 2019	Scalia et al., 2019	Harada et al., 2021	Chen et al., 2022	Ahmed et al., 2024	Alasiri et al., 2023	Meunier et al., 2023	He et al., 2024	Hill et al., 2024	Gani et al., 2025
People domain													
Negative impact on physician/patient relationship	Risk assessment tools are considered intrusive and disruptive to physician/patient communications.		●				●		●	●	●		●
HCPs lack knowledge & skills	HCPs demonstrate poor understanding of AI & medical informatics, & lack the skills requires to incorporate risk assessment into routine practice.		●		●	◍		◍		●		●	●
Negative perceptions towards tools	HCPs & patients perceive tools as time consuming, unable to account for variation between patients & lacking holistic perspective.		●	●	●		●	◍	●	●		●	●
Lack of integration with EHR & clinical workflows	HCPs report alert-fatigue, generation of additional tasks & inopportune timing of pop-ups. Many tools are reliant on potentially erroneous manual data entry & are not integrated with EHR or wider systems.	○	●	●	●	◍	●	◍	●	●		●	●
Overly complex outputs	Risk assessment outputs are too complex for patients.				●						●		
System													
Incompatibility with continuity of care	Uncertain how to share data between HCPs & healthcare settings.									●	●		

Technical issues & glitches	IT hardware is lacking & tools are slow or glitchy.						●		●	●			●
Design domain													
Outputs are not clearly visible or available immediately	Outputs are not always actionable within the appointment.		●				●						
Poor presentation & layout	Poor formatting that lacks graphical representations of risk.		●		●		●			●	●		●
Not intuitive or user-friendly	Tools require too much user input & lack standardised terminology.	○	●				●			●			
Risk domain													
Algorithmic decision-making lacks transparency	HCPs have concerns over the validity, reliability, accuracy & security of risk assessment tools. Algorithmic decision making is seen as a 'black box'.			●		⊜		⊜					●
Input data is incomplete & poor quality	EHR is incomplete & of poor quality, the use of historical data in risk modelling is challenging.						●	⊜		●			
Tools are not updated & maintained over time	Ongoing technical support is needed to support implementation & sustain routine usage.	○										●	●
Lack of transparent legal & regulatory frameworks	Lack of clear regulation and legal implications. Unclear where liability sits & a lack of national guidelines.							⊜			●	●	
Competing vendors for AI platforms	Concerns that AI vendors are motivated by profit rather than patient care.							⊜					

AI – artificial intelligence; EHR – electronic health records; IT – information technology; HCP – healthcare professional.

Filled circle: low risk of bias; clear circle: high risk of bias; stiped circle: unclear risk of bias
